# Supplementary material for: BayesAge 2.0: a maximum likelihood algorithm to predict transcriptomic age
Source: GeroScience. 2025 Jan 3;47(3):5389–99. doi: 10.1007/s11357-024-01499-0 (PMC12181495; doi:10.1007/s11357-024-01499-0)
Supplement: Supplementary file 1 — Supplementary file1 (DOCX 14 KB) [file 11357_2024_1499_MOESM1_ESM.docx]

**Supplementary Information**

The best α and λ hyperparameter used for the elastic net clocks are included in the supplementary table below:

| **Tissue** | **Alpha ( α)** | **L1 Ratio (λ )** | **MAE** |
| --- | --- | --- | --- |
| BAT | 0.001 | 1.0 | 2.489 |
| Bone | 0.01 | 0.3 | 3.906 |
| Brain | 0.01 | 1.0 | 2.190 |
| GAT | 0.01 | 0.6 | 2.040 |
| Heart | 0.01 | 1.0 | 4.829 |
| Kidney | 0.001 | 0.8 | 2.338 |
| Limb Muscle | 0.01 | 0.9 | 2.468 |
| Liver | 0.01 | 1.0 | 2.121 |
| Lung | 0.01 | 1.0 | 2.265 |
| Bone Marrow | 0.01 | 0.3 | 3.906 |
| MAT | 1.0 | 1.0 | 2.964 |
| Pancreas | 0.1 | 1.0 | 3.398 |
| SCAT | 0.001 | 0.9 | 3.069 |
| Skin | 0.01 | 1.0 | 2.965 |
| Small Intestine | 0.1 | 1.0 | 1.899 |
| Spleen | 0.001 | 1.0 | 2.558 |
| WBC | 0.01 | 0.6 | 2.496 |
